# Various Configurations of Au@Pt Nanostructures on Modified Electrochemical Sensors for H_2_O_2_ Detection

**DOI:** 10.1021/acsanm.5c03116

**Published:** 2025-07-22

**Authors:** Bahar Mostafiz, Johanna Suni, Edna De Jesus Cabrera, Nidhin George Mathews, Rituporn Gogoi, Gaurav Mohanty, Vipul Sharma, Emilia Peltola

**Affiliations:** † Department of Mechanical and Materials Engineering, 8058 University of Turku, Turku FI-20014, Finland; ‡ Materials Science and Environmental Engineering, Faculty of Engineering and Natural Sciences, 528748 Tampere University, Tampere FI-33014, Finland

**Keywords:** hydrogen peroxide (H_2_O_2_), Au@Pt nanorods, Pt oxidation state, nanoparticle fabrication, electrochemical sensor, surface chemistry, cell toxicity assay

## Abstract

Hydrogen peroxide (H_2_O_2_) is a vital metabolite involved in numerous biological processes, with physiological concentrations in humans ranging from 1 to 50 μM. Its rapid production, utilization, and decomposition make accurate low-concentration detection challenging. Although precious metals such as gold and platinum are effective for H_2_O_2_ detection, their high cost and limited availability necessitate alternative strategies. Nanostructuring these materials into core–shell nanorods (their size ∼ 40 nm in length) offers a sustainable, efficient solution by reducing material usage while enhancing performance. In this study, we modified glassy carbon electrodes with two types of Au@Pt nanorods (NR) for H_2_O_2_’s cyclic voltammetric and chronoamperometric detection: plain-surfaced (Smooth) and appendaged-surfaced (Hairy). Both sensors exhibit rapid stabilization, achieving reliable measurements within 5 s, suitable for capturing the volatile nature of H_2_O_2_. The Hairy NRs demonstrate superior performance, attributed to the increased presence of catalytically active Pt(0) compared to the less active Pt­(II) in Smooth NRs. This difference in oxidation states, combined with the enhanced surface geometry of Hairy NRs, results in faster kinetics, a wider linear detection range (500 nM–50 μM vs 1–50 μM), lower detection limit (189 nM vs 370 nM), and nearly double sensitivity. To simulate physiological conditions, we assessed oxygen interference and evaluated performance in biologically relevant environments. Cell viability tests were conducted to determine the nanoparticles’ toxicity toward neuroblastic cells. These findings support further development of modified Au@Pt nanorod electrodes for in vivo and in vitro applications. With rapid response times, favorable detection limits, and high sensitivity, these sensors are promising for biomedical diagnostics, environmental monitoring, and studying neurotransmitters like glutamate.

## Graphical abstract

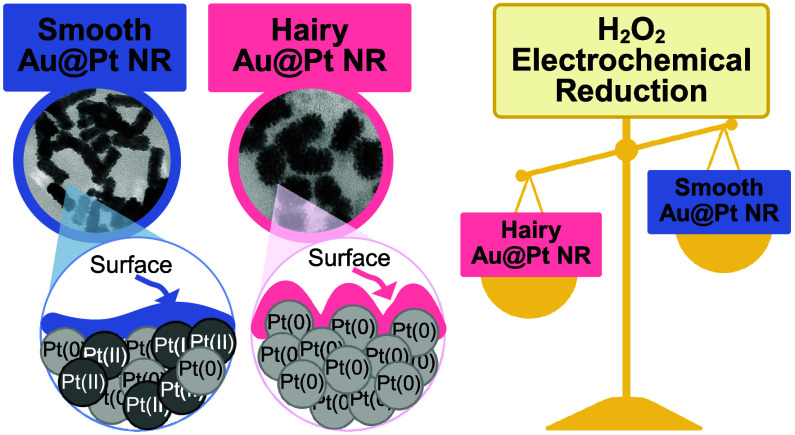

## Introduction

Hydrogen peroxide (H_2_O_2_) is a metabolite that is extensively involved in redox metabolic reactions and processes within cells across various species, including bacteria, plants, and mammals.^1^ Particularly, it is recognized as a key molecule in detection, modulation, and signaling associated with redox metabolism. H_2_O_2_ can easily diffuse through intercellular spaces,^2^ impacting a wide range of cellular activities. Its critical roles in these biological activities can be modulating transcription activity,^1^ regulating mechanisms to adapt to various environmental stressors,^3,4^ and enhancing cellular signaling.^1^ The normal range of H_2_O_2_ in plasma is a topic of ongoing debate, with some studies suggesting a range of 1–5 μM, supported by sensitive techniques such as chemiluminescence and microfluidic devices, while others report much higher values (reaching to 50 μM) in inflammatory conditions, attributed to increased production by phagocytes and endothelial cells in response to inflammation.^5^ H_2_O_2_ in plasma is primarily generated by several sources, of which NADPH oxidases (NOXs) and xanthine oxidase are well-known. In addition, the autoxidation of small molecules (e.g., ascorbate and glutathione) can generate H_2_O_2_ in the presence of transition metals.^5–7^ In biosensing, some oxidase enzymes, such as glucose and glutamate oxidase, also produce H_2_O_2_.^8–10^ Measuring H_2_O_2_ in plasma is technically challenging due to (a) compartmentalization within specific regions, (b) the existence of concentration gradients influenced by antioxidants and enzyme activity, and (c) interfering factors, such as ascorbate and uric acid. All of these can lead to inaccurate estimation of the true concentration.^11,12^ Additionally, some methods such as titration or spectrophotometry may not be sensitive enough to accurately detect low concentrations of H_2_O_2_ in plasma. The mentioned traditional methods are often laborious and time-consuming, and their analytical reagents can be expensive. Electrochemical methods for H_2_O_2_ detection have gained significant interest in both academic and clinical settings. They offer distinct advantages for studies of H_2_O_2_-related neurological impacts over the traditional methods, particularly in terms of cost-efficiency and in vivo compatibility. The inherent electroactivity of H_2_O_2_ facilitates its direct detection, simplifying the measurement process and reducing the need for additional chemicals.

The use of nanoparticles (NPs) like gold and platinum has proven to be an effective electrode modification for H_2_O_2_ reduction, with both metals exhibiting a high affinity toward H_2_O_2_.^13–15^ Compared to fabricating the entire electrode from these precious metals However, producing NPs vastly reduces the total amount of metal used. This is crucial for sustainability, especially given the scarcity of these materials.^16^ Also, when a higher percentage of an electrode is made from nanoparticles, its biocompatibility improves. This improvement occurs because the nanoparticles better match the mechanical properties of biological tissue, enabling more seamless integration with the cellular environment compared to the rigid nature of bulk-material electrodes.^17^

Gold NPs (Au NPs) are widely used due to their exceptional catalytic activity and ease of functionalization, which enhance conductivity and sensitivity.^18^ Similarly, platinum NPs (Pt NPs) are essential for their improved sensor sensitivity and stability, making them valuable in clinical diagnostics and neurological research.^15^

Core–shell particles offer significant benefits including enhanced stability and dispersion, customizable surface functions, and improved functionality through surface modifications and core release control.^19–22^ However, it is challenging to incorporate them as electrochemical modifiers because, due to hardships of controlling the size and shape of the final particles, they can introduce heterogeneity in the active sites of the surface, reducing the sensor’s repeatability.^21^

It is crucial to understand that these nanoparticles can show different catalytic behaviors in various orientations, growth directions, morphologies, and oxidation states. However, there are very limited studies conducting comprehensive studies on these criteria all at once that investigate the impact of these variations on the electrochemical activities of modified sensors. This study focuses on nanoparticles (size ∼ 40 nm in length) with identical chemical composition but carefully controlled and intentionally varied structures, allowing a direct comparison of how morphology, such as shape, facet orientation, and crystallinity, influences the intrinsic electrochemical behavior.

## Experimental Section

### Reagents

The synthesis of Au NPs involved using cetyltrimethylammonium bromide (CTAB), gold­(III) chloride trihydrate (HAuCl_4_·3H_2_O), silver nitrate (AgNO_3_), sodium chloride (NaCl), l-ascorbic acid (AA), and sodium borohydride (NaBH_4_). For the fabrication of Au@Ag NRs, polyvinylpyrrolindone (PVP) and sodium hydroxide (NaOH) were obtained. Additionally, potassium tetrachloroplatinate­(II) (K_2_PtCl_4_) and sulfuric acid (H_2_SO_4_) were used for producing Au@Pt NPs. Salt (NaCl) and disodium hydrogen phosphate (Na_2_HPO_4_), as well as potassium chloride (KCl) and monopotassium phosphate (KH_2_PO_4_) were dissolved in Mili-Q water to form the phosphate buffered saline (PBS) solution with a final pH of 7.4. Electrochemical studies were carried out by using H_2_O_2_, Hexaamineruthenium­(III) chloride ([Ru­(NH_3_)_6_] Cl_3_), and Potassium hexacyanoferrate­(III) (K_3_[Fe­(CN)_6_]). All of the above chemicals were acquired from Sigma-Aldrich, except for (KCl) and (KH_2_PO_4_), which were acquired from VWR. All chemicals were used as received. All stock solutions for nanoparticle fabrication were prepared in MiliQ water and all the rest of the solutions were prepared in RO water. All measurements were done at room-temperature

### Apparatus

Electrochemical measurements were conducted using a Gamry Reference 620 (Warminster, PA, USA) potentiostat in a three-electrode setup featuring an Ag/AgCl reference electrode (CH Instruments), a platinum wire counter electrode, and a 3 mm diameter glassy carbon working electrode from redox.me. Prior to every measurement, the working electrode was polished with a diamond monocrystalline suspension 1 μm (MetaDi, Buehler) and rinsed with MiliQ water. Centrifugation was performed with an Eppendorf model 5810 centrifuge with accommodations for 50- and 15 mL Falcon tubes. Electron microscopy analysis was carried out using a Jeol JEM-1400Plus TEM at 80 kV by depositing the nanorods on to a copper grid. High resolution transmission electron microscopy (HR-TEM) was performed using a JEOL JEM-F200 TEM at 300 kV. The UV–vis spectrometer was an Analytik Jena model SPECORD 200 PLUS Double-beam spectrophotometer. X-ray photoelectron spectra (XPS) were collected using Thermo Scientific Nexsa surface analysis with an Al kα source (1486.7 eV). All the spectra were acquired with a spot size of 400 μm, and dual-charge compensation was applied. Cell viability tests were collected on a Tecan Infinite F 200 Pro plate reader.

### Particle Fabrication and Electrode Modification Procedure

The production of Au@Pt core–shell nanorods was based on a method outlined in a previous study^23^ (Figure 1), which can be found in the Supporting Information.

**Figure 1:**
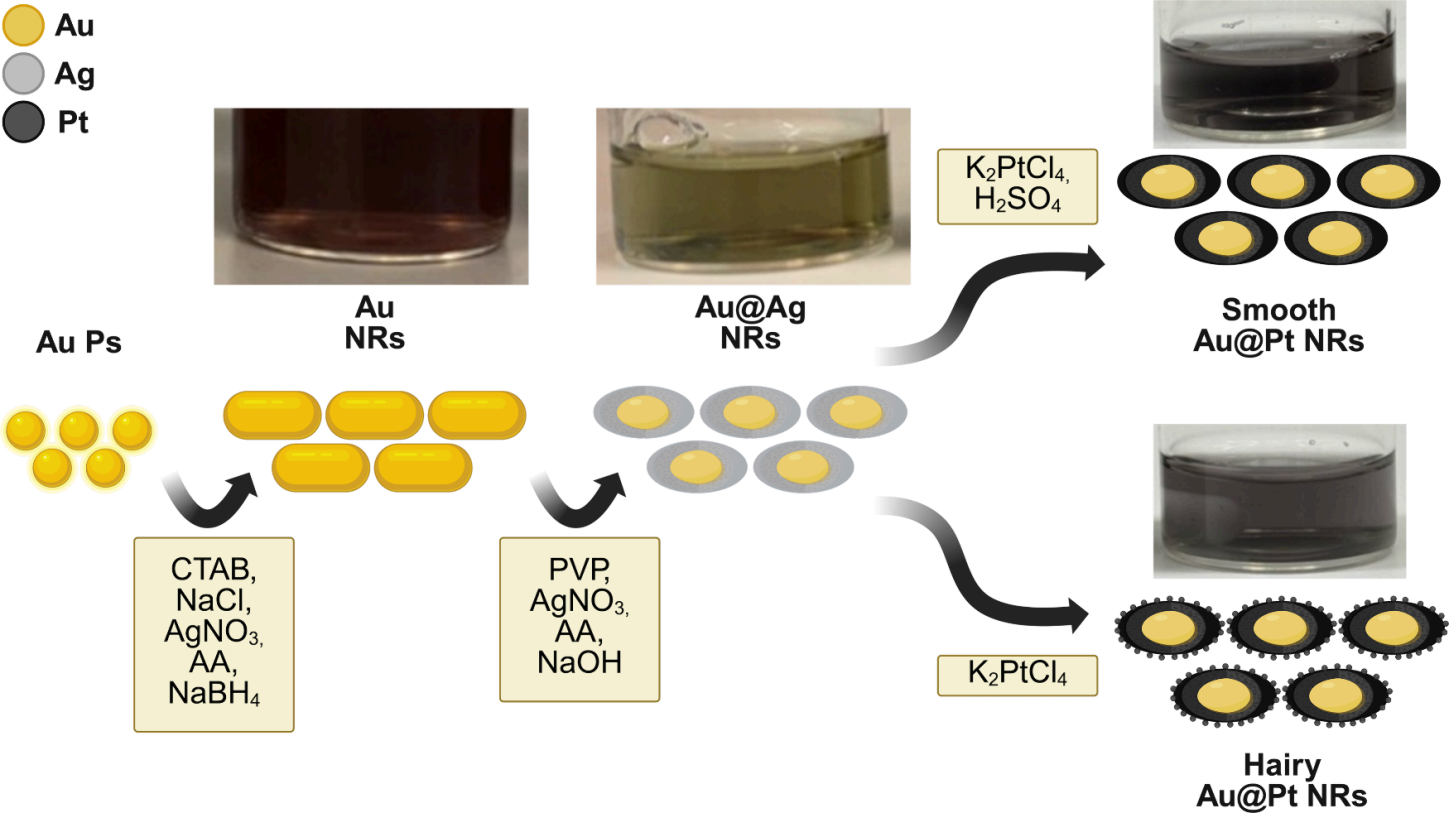
Au@Pt core–shell nanorods fabrication procedure (Created in BioRender. Mostafiz, B. (2025)).

After fabrication, the Smooth and Hairy Au@Pt solutions were drop-casted using a 20 μL micropipette to deposit 5 μL of the mentioned solutions on a polished glassy carbon electrode and left to dry for 2 h in the dark at room temperature.

### Electrochemistry

To investigate the electrochemical characteristics of the unmodified and modified glassy carbon electrodes, we prepared several solutions were prepared. Each data point was acquired between 3 and 4 times. In most measurements, three electrode types (unmodified, Smooth Au@Pt, and Hairy Au@Pt modified) were compared to investigate the effects of the nanoparticles on their electrochemical performance.

Cyclic voltammetric (CV) studies were carried out for several purposes. However, in all of them, cyclic voltammograms ran for 3 cycles.

Potential window or water window refers to the general performance range of a sensor in a given electrolyte, i.e., if a molecule is reacting in potential X, is the sensor stable enough in the mentioned potential to not engage in oxygen or hydrogen evolution reactions. It is defined using a self-chosen threshold current value. In this paper, the threshold intensity was kept to ≈1 μA (nearly 10^2^ times higher than baseline intensity). The water window potential range was determined for the unmodified and modified electrodes in PBS (pH 7.4) solutions. Following this, electrode performance was evaluated by using an outer-sphere redox (OSR) probe, Ru­(NH_3_)_6_^3+/2+^, and an inner-sphere redox (ISR) probe, Fe­(CN)_6_^3–/4–^. ISR probes, like Fe­(CN)_6_^3–/4–^, operate through direct interaction with the electrode surface, often forming transient coordination bonds, making it highly sensitive to surface properties, such as morphology and metal impurities, which in turn offer detailed insights into surface modifications and chemistry. In contrast, the OSR probe, such as Ru­(NH_3_)_6_^3+/2+^, manifesting response through electron tunnelling without direct contact, provide information on reaction kinetics, diffusion behavior, and broader solution-phase dynamics rather than surface-specific characteristics.^24^ The OSR probe, a 1 mM Ru­(NH_3_)_6_^3+/2+^ in 1 M KCL solution, was tested within a potential range of −500 to 200 mV, while the ISR probe solution was prepared as a 2 mM Fe­(CN)_6_^3–/4–^ solution in 0.1 M KCl and studied in a potential range of −100 to 500 mV. Both probes were tested at a scan rate of 50 mV·s^–1^.

Electrochemical impedance spectroscopy (EIS) is a method used to study how electrons move and how charges are transferred at the boundary between the electrode and the electrolyte.^25^ It was performed to understand these interactions in a 2 mM Fe­(CN)_6_^3–/4–^ solution in 0.1 M KCl solution at a frequency range of 1 Hz to 1 MHz at a set open-circuit potential based on the modification of the sensor.

Before the H_2_O_2_ CVs were obtained, preliminary cycles in PBS (pH 7.4) were done to establish a stable baseline. Next, the PBS solutions were spiked by H_2_O_2_ stock solutions and then stirred in the cell for 10 s before the measurements started. Subsequently, biological concentrations of H_2_O_2_ in PBS were measured in the electrode’s potential window with a scan rate of 50 mV·s^–1^. Additionally, the relation between the peak intensity and the scan rate was tested in scan rates 200 to 2 in 50 μM H_2_O_2_/PBS solutions.

The effect of the presence of O_2_ on the sensor performance was studied by comparing the CV peaks of PBS and 50 μM H_2_O_2_ solutions with or without bubbling the solutions with a pressured air inlet containing 21% of the O_2_ gas for 15 min. The peaks were obtained immediately after the gas inlet was turned off in a sealed cell in the same potential windows of the corresponding electrode at a scan rate of 50 mV·s^–1^.

Chronoamperometry (CA) was subsequently used to study the linearity of peak intensity growth as the concentration of H_2_O_2_ in PBS increased. The spiked H_2_O_2_ solution was stirred for 10 s before the measurements started. First, the step potential was optimized based on the signal-to-noise ratio in detecting PBS and 50 μM H_2_O_2_ solutions, respectively. The applied potential ranged from 300 to 700 mV. To ensure stability for data analysis, CA values were averaged between 3 and 3.5 s. While increasing the potential may enhance current intensity, it also elevates the capacitive current, thereby increasing the noise levels. To normalize the response efficacity, background signals were recorded across the same potential range, and the signal-to-noise (S/N) ratio was calculated. Once the optimization was complete, the starting potential was set to 200 mV versus an Ag/AgCl reference for 1 s, followed by a step to 500 mV to measure the oxidation peak intensity. The entire measurement lasted a total of 51 s. The concentration addition was started from 500 nm and then spiked to 1 μM and then increased by 1 μM steps until 5 μM, then the concentration steps were increased to 5 μM until they reached concentration was 20 μM and from then on until 50 μM the added units were 10 μM.

### Cell Viability Tests

Cells were cultured in a humidified incubator with 5% CO_2_ in the air. SH-SY5Y neuroblastoma cells (Cytion) were cultured in Dulbecco’s Modified Eagle Medium/Nutrient Mixture F-12, no phenol red (Thermo Fisher) supplemented with 15% fetal bovine serum (FBS) and antibiotics, 100 IU/mL of penicillin and 100 μg/mL of streptomycin. 500 μL of Smooth and Hairy Au@Pt NRs solutions were transferred into Eppendorf 2 mL tubes and centrifuged for 5 min in 13000 rpm. The surfactant solution on top was extracted as much as possible using pipettes and collected in a separate Eppendorf 2 mL tube. Next, 500 μL MilliQ water was spiked on the centrifuged NRs and mixed. Then, NRs solutions were transferred to a 48-well plate together with the cells. The seeding density of SH-SY5Y cells was 30,000 cells/well. The toxicities of Smooth and Hairy NPs were determined at different concentrations (2.5, 5.0, and 7.5 μL of NRs solutions, volume of the well was 200 μL). In addition to NRs, the toxicity of surfactant was tested by spiking 5 μL of surfactant solution in corresponding wells. Triplicates were made for all concentrations. The plate was incubated for 24 h at 37 °C in a 5% CO_2_ atmosphere.

3-(4,5-dimethylthiazol-2-yl) 2,5-diphenyltetrazolium bromide (MTT) assay was utilized to evaluate the viability of cells grown on Smooth and Hairy NRs samples. Five mg/mL of MTT (Sigma-Aldrich) was added in the medium (final concentration 0.5 mg/mL). The samples were incubated for 3 h at 37 °C in a 5% CO_2_ atmosphere. MTT crystals were dissolved by adding 10% SDS in 1 mM HCl (1:2) to the wells and incubating the well-plate at 37 °C overnight. Absorbance was measured at 570 nm, and data was collected from triplicate samples using an automated plate reader (Tecan).

### Data Analysis

HR-TEM images were analyzed with Fiji software by using the inverse FFT method. In XPS data, the high-resolution core level spectra were deconvoluted using CasaXPS Version 2.3 as a curve fitting software using smart background (modified Shirley background uses additional constraints to keep background intensity less than the actual data at any point in the region). All of the spectra were corrected for charge-shift with reference to the adventitious carbon (C–C component) positioned at 284.8 eV.

All electrochemical data were analyzed by using Gamry Echem Analyst 2 and Python. Statistical and data visualization were done in Python as well. The sensitivity of calibration, which reflects the electrode’s responsiveness to variations in H_2_O_2_ concentration, was determined from the slope of the calibration curve and is reported in microamperes per micromole (μA/μM). The detection limit (LOD) was computed using the equation (LOD = 3*sd/*m*), where *sd* represents the standard deviation of the blank sample measurements, and *m* denotes the calibration sensitivity.

The relative cell viability (%) was calculated using the following formula, where *A_sample_* represents the absorbance of the test samples, and *A_control_* represents the absorbance of the control sample, which consisted of cells without nanoparticles (eq 1).

1 $$\mathrm{Cell viability}\left(\%\right)=\left(\frac{A_{sample}}{A_{control}}\right)\times 100$$

The data are displayed as the average value based on three or more independent experiments (*N* ≥ 3).

## Results and Discussion

### Morphological Characterization

#### TEM Characterization

When comparing the TEM images of the Smooth and Hairy Au@Pt NPs, most particles were found to be nearly 40 nm in length (Figure 2 and Figure S1). The overall dimensions of the core–shell particles are comparable to those of Au NRs. However, noticeable structural differences exist between them. In comparison to the Au NRs (Figure 2a) particles, the Smooth Au@Pt NRs (Figure 2b) have a wrinkled surface that shows an increase in the surface area. This is the result of the tight encapsulation of the Au core by the Pt shell, suggesting that the bonding occurs directly on the surface. In contrast, the outer layer of the Hairy Au@Pt NRs (Figure 2c) is visibly rougher, characterized by homogeneous hair-like appendages growing in every direction. These appendages drastically increase the surface roughness and create valley-like structures around the shell, which could provide spaces for analyte molecules to diffuse into and establish bonds.

**Figure 2:**
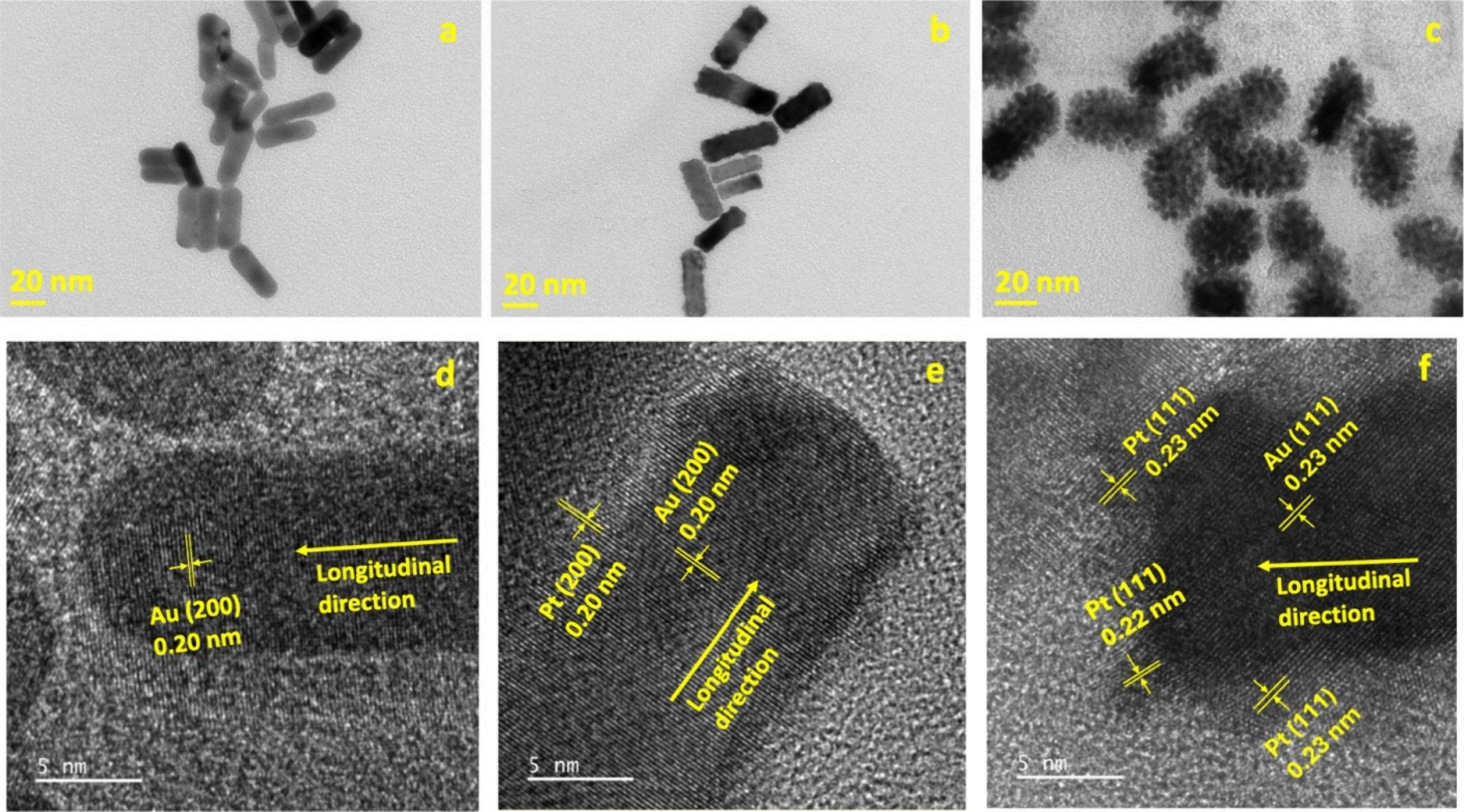
Bright field TEM images of (a) Au NRs, (b) Smooth Au@Pt NRs, and (c) Hairy Au@Pt NRs. HR-TEM images of (d) Au NRs, (e) Smooth Au@Pt NRs, and (f) Hairy Au@Pt NRs with the clearly observed lattice planes indexed.

A deeper structural analysis was conducted by using HR-TEM imaging. From the TEM observations, Au nanorods were observed to be single crystals with fcc lattice in both Smooth and Hairy Au@Pt NRs. The Au and Pt atomic layers seem to have perfect lattice matching with each other which suggests epitaxial growth of Pt on Au in both samples (Figure 2e and 2f). This is expected as the lattice mismatch between Au and Pt is less than 4%, which is favorable for the epitaxial growth conditions. In Smooth Au@Pt NRs, *d*-spacing for both Au and Pt layers was calculated to be ∼0.20 nm, which corresponds to the (200) lattice plane along the longitudinal axis direction of the nanorod, which is the longitudinal direction. In the case of Hairy Au@Pt NRs, *d*-spacing of the visible planes was calculated to be ∼0.23 nm, which corresponds to the (111) lattice plane. The inclination of this set of planes with respect to the longitudinal direction is closer to the theoretical angle between the (111) and (200) lattice planes. This confirms that the longitudinal direction for the Hairy Au@Pt NRs is also (200). In Addition to Au@Pt NRs, the lattice planes for Au NRs were calculated to be (200) (Figure 2d). For both Smooth and Hairy Au@Pt NRs, epitaxial growth of Pt on Au was observed, with lattice planes matching between both. These data was further confirmed by comparing them to the previous study.^23^

#### UV–vis and XPS Studies

The characteristic UV–visible absorption spectrum of core–shells suggests successful formation, as shown in Figure 3a. First, the absorption of the Au particles deep yellow solution, is negligible in the measured spectrum of 400 to 1200 nm. When these particles undergo the first fabrication step to form Au NRs, the purple-brownish solution’s absorption shows two peaks, one at 515 nm (indicative of transverse plasmon resonance) and another in the near-infrared region at 783 nm related to longitudinal plasmon resonance. Moving on to the second step, upon the formation of Au@Ag core–shells, the UV–vis peaks have now shifted to 505 and 696 nm, respectively. This shifting may be attributed to the variations in dielectric functions between silver and gold, and a reduction in the aspect ratio might also contribute to the shift. In Au@Pt NRs, there was a quenching of longitudinal plasmonic absorption bands, indicating a coverage of the Au NRs by Pt.

**Figure 3:**
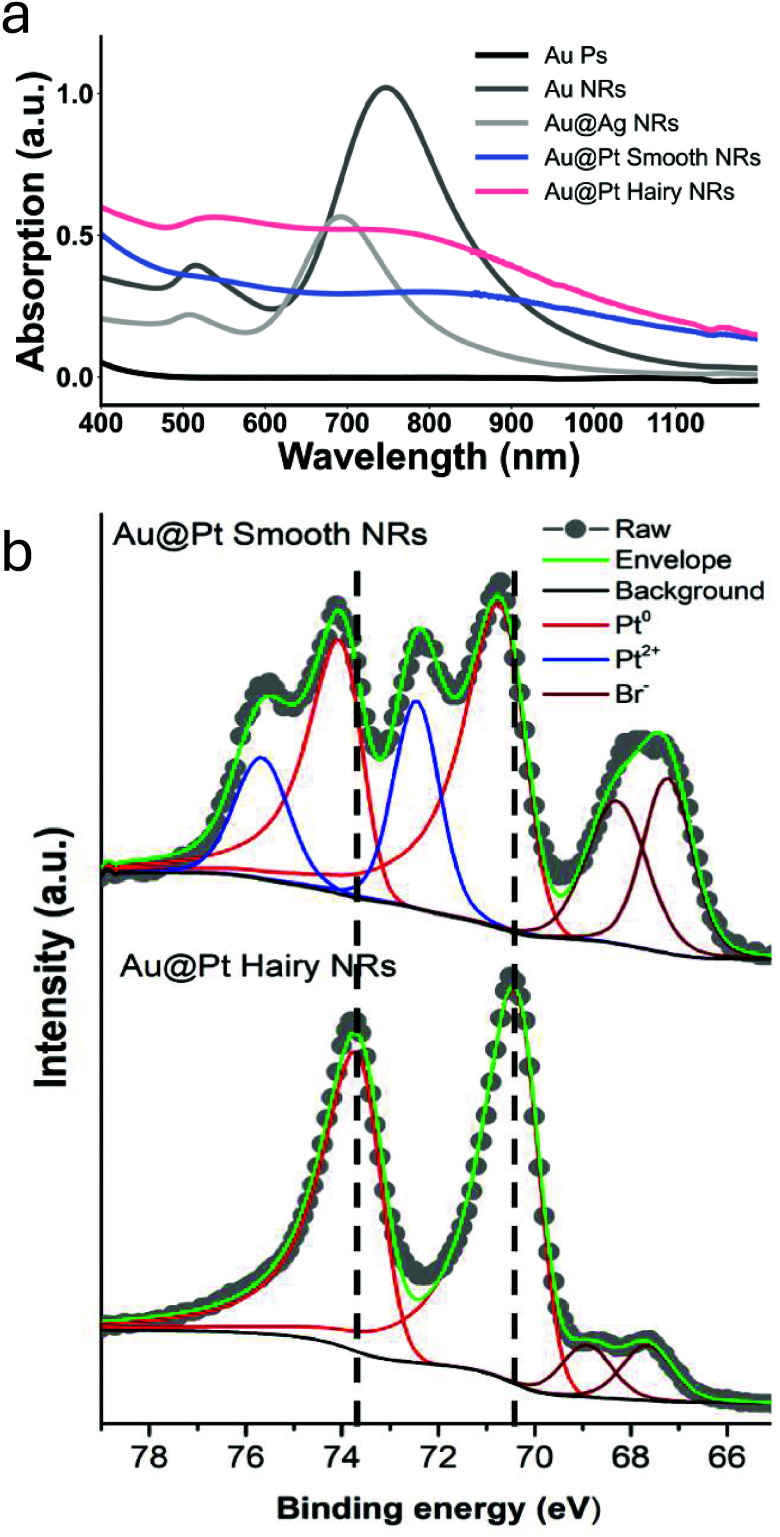
(a) UV–vis absorption spectra of Au particles (black), Au NRs (dark gray), Au@Ag NRs (light gray), Smooth Au@Pt NRs (blue), and Hairy Au@Pt NRs (pink). (b) Comparing XPS narrow scan spectra of Pt 4f and Br 3d for Smooth and Hairy Au@Pt NRs.

The UV–vis spectrum of Hairy Au@Pt nanorods exhibits a weak absorption peak near 515 nm, attributed to the transverse plasmon resonance of the Au core. This is due to UV–visible spectroscopy’s sensitivity to subtle changes in the local dielectric environment. For Smooth Au@Pt NRs, a conformal Pt shell uniformly coats the Au surface, effectively quenching the Au plasmon resonance and resulting in complete suppression of the characteristic Au peak. While, Hairy Au@Pt NRs exhibit a different growth mechanism, where Pt forms branched structures that lead to a nonuniform shell. This directional growth results in regions with ultrathin Pt coverage, especially at branch bases or junctions, allowing a partial interaction of light with the Au core and giving rise to the observed weak absorption at ∼515 nm.

To verify the elemental composition of Au@Pt NRs, XPS results were acquired. As shown in Figure S2, the XPS survey spectra exhibit distinct peaks characteristic of Au and Pt in both Smooth and Hairy Au@Pt NRs, along with evidence of CTAB presence (C 1s, O 1s, N 1s, and Br 3d). High-resolution narrow scans of the characteristic elements, normalized to Pt 4f peaks (Figure 3b), further reveal that the Hairy Au@Pt NRs display spin–orbit doublets peaks at 70.4 and 73.7 eV (Δ*E* ∼ 3.3 eV), corresponding to Pt 4f_7/2_ and Pt 4f_5/2_, respectively. These asymmetric peaks indicate a Pt^0^ oxidation state. Although the spectra for Smooth Au@Pt NRs are very similar, important distinctions are evident. This shift occurs in the Pt 4f region: the Pt^0^ peaks for the Smooth Au@Pt NRs lie ∼0.4 eV higher in binding energy than the corresponding Pt^0^ peaks in the Hairy Au@Pt NRs, indicating an overall upshift of the Pt 4f spectral lines.

The shift in binding energy may arise from several interrelated factors, including the mass differences of Pt atoms surrounding the Au core and variations in bonding energies due to differences in atomic coordination between the two species.^26,27^ Another reason can be related to their respective particle sizes. In the Smooth Au@Pt NRs, the Pt shell is compact and aligned with the Au core, forming a continuous and dense structure. This morphology does not significantly increase the overall particle size, suggesting that Pt atoms form smaller, more tightly packed structures. In contrast, the Hairy Au@Pt NRs exhibit a branched growth pattern, where Pt structures extend outward from the core surface into the surrounding space. This promotes the formation of larger, less densely packed Pt structures, as confirmed by HR-TEM imaging (Figure 2e and 2f). The reduced size of nanoparticles in Smooth Au@Pt NRs was observed due to the controlled blocking of Pt deposition by bisulfate ions, as reported in our previous work.^23^ Notably, the inverse relationship between nanoparticle size and core-level binding energy has been reported in several prior studies.^28–30^ Additionally, differences in growth kinetics may contribute to the observed variation and the following shift in the binding energy: the rapid formation of the branched Pt shell in the Hairy Au@Pt NRs favors the stabilization of metallic Pt^0^, while the slower, more controlled growth in the Smooth Au@Pt NRs facilitates partial oxidation, resulting in the presence of Pt^2+^. Bringing us to consider a dominant contributor to the observed binding energy differences which is the oxidation states of Pt. Particles containing a larger fraction of ionic Pt (e.g., Pt^2+^) exhibit noticeably higher Pt 4f binding energies in comparison to samples that are predominantly metallic. The more ionic environment withdraws electron density from the Pt centers, reduces final-state screening, and thereby elevates the core-level energy, making oxidation state a decisive factor alongside size, shell thickness, and interfacial bonding.

In terms of potential sensing behavior, the higher oxidation state in the Smooth Au@Pt NRs may indicate stronger surface adsorption of ligands (e.g., Cl^–^, NH_3_) or intermediates, leading to electrochemical fouling of the electrode surface and reducing the surface reactivity. This behavior is likely due to the presence of Pt^2+^ species, which, unlike catalytically active Pt^0^, are consumed during the reaction. In contrast, the more stable oxidation state (Pt^0^) found in the Hairy Au@Pt NRs (characteristic of metallic Pt) may facilitate more efficient interaction with H_2_O_2_, lowering the activation energy required for H_2_O_2_ bond decomposition and thereby enhancing sensitivity.^23,31^

Interestingly, another observation from the XPS analysis concerns the surfactant peaks. The presence of CTAB is confirmed by the Br peak, which appears lower in the Hairy Au@Pt NRs structure compared to the Smooth Au@Pt NRs structure. We hypothesize that this difference arises because the Hairy Au@Pt NRs more firmly trap CTAB molecules with their complex appendages and defective surfaces. Consequently, when the electron beam interacts with these structures, it encounters a densely packed environment where the 5–10 nm penetration is insufficient to accurately resolve the composition—likely due to the reabsorption of secondary electrons.

This hypothesis is further supported by observations from the HR-TEM session. Despite multiple washing steps, imaging the Hairy Au@Pt NRs resulted in fast sample burning and a limited acquisition time for high-quality images. We believe this behavior was due to the higher carbon content—originating from CTAB—present in the Hairy structures, a problem not encountered with Smooth Au@Pt NRs.

### Electrochemical Studies

#### Electrochemical Characterization

As illustrated in Figure 4a, the unmodified electrode has the widest potential window in PBS (from −200 to 900 mV), compared to the modified electrodes. The Smooth Au@Pt modified electrode has a potential range from 0 to 800 mV, whereas the Hairy Au@Pt modified electrode shows a slightly narrower window (0 to 700 mV). This can be attributed to the difference in structure of the nanoparticle surface and therefore catalytic behaviors. If there is more surface available for the absorption, it facilitates the formation of a monolayer of water more, resulting in narrower potential windows.

**Figure 4:**
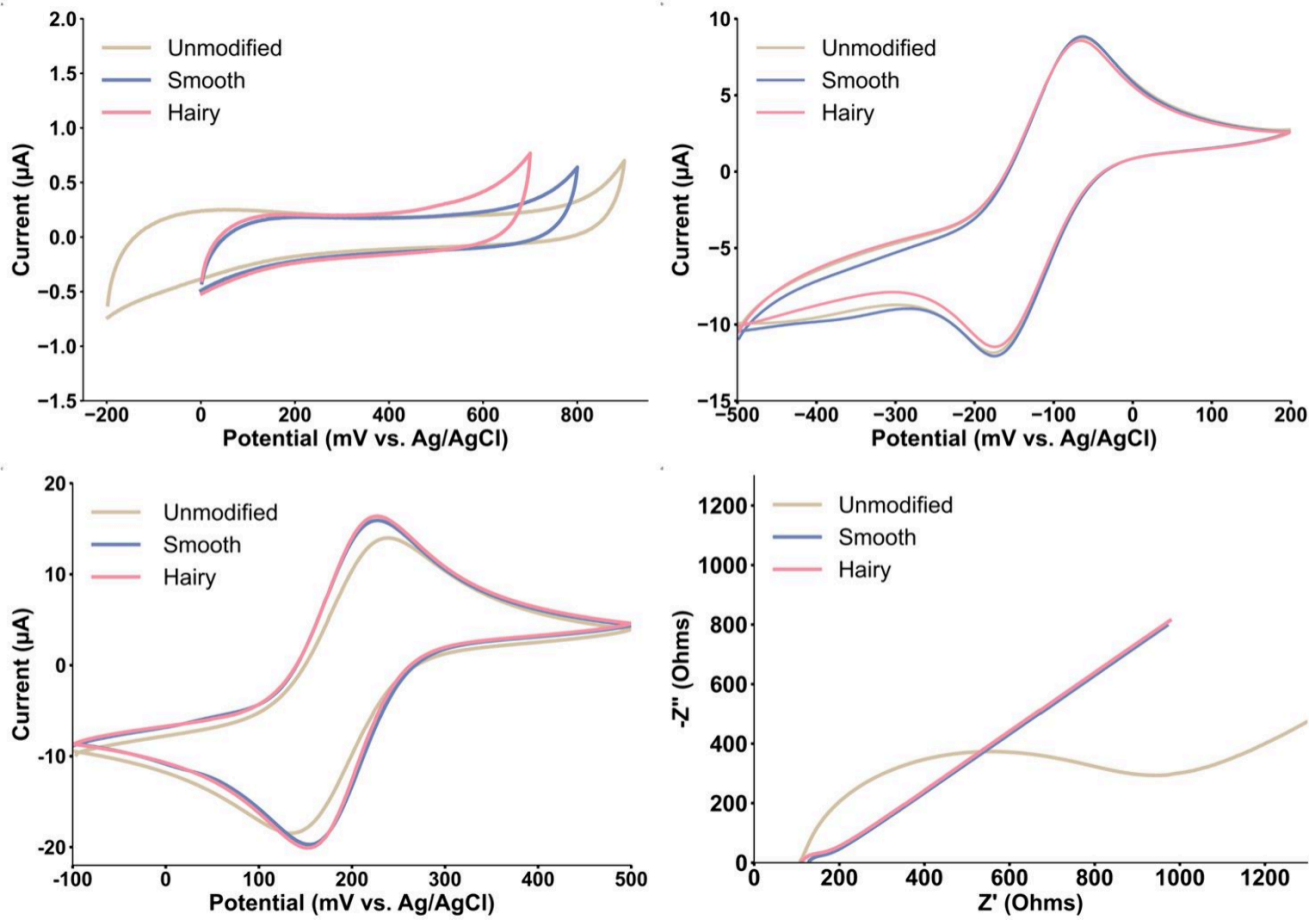
Cyclic voltammogram of (a) the potential window obtained in PBS, (b) the OSR probe obtained in Ru­(NH_3_)_6_^3+/2+^ 1 mM/KCl 1 M, (c) ISR probe obtained in Fe­(CN)_6_^3–/4–^ 2 mM/KCl 0.1 M, all at the scan rate of 50 mV·s^–1^, and (d) EIS Nyquist plot obtained in Fe­(CN)_6_^3–/4–^ 2 mM/KCl 0.1 M, at a frequency range of 1 Hz to 1 MHz on the unmodified (cream), Smooth Au@Pt NRs (blue), and Hairy Au@Pt NR-modified (pink) electrodes.

Next, the electrochemical response of Ru­(NH_3_)_6_^3+/2+^(OSR) is shown (Figure 4b). It can be seen that the modification of the electrode surface with metallic NPs has not significantly changed the electron transfer behavior against the OSR probe, as the Δ*E*_*P*_ and the anodic peak current intensities of all three electrodes are in the same range (unmodified: 114 ± 4 mV, 8.7 μA; Smooth Au@Pt: 113 ± 1 mV, 8.9 μA; Hairy Au@Pt: 109 ± 1 mV, 8.6 μA). These similarities are reflected in current density (*j*) values that is calculated to be 1.2 μA.mm^–2^ for all three electrodes. These values, some observed before in previous studies (same delta E separation vs glassy carbon)^32–35^ suggest a slow kinetic reaction or possible diffusion limitations.

In Figure 4c, we are examining the response toward Fe­(CN)_6_^3–/4–^ probe (ISR). Compared to the unmodified electrode, the current intensities of modified electrodes are approximately 14% and 17% higher for Smooth and Hairy Au@Pt modified electrodes, respectively. This increase indicates a relatively higher rate of electro-oxidation of Fe^3+/4+^ on the core–shell-modified electrodes that points to noticeable changes in the surface properties of the electrode. Also, there is a notable Δ*E*_*P*_ shift from the unmodified electrode to the modified ones. The peak-to-peak potential separation has improved on the modified electrodes vs the ISR probe. The Δ*E*_*P*_ for the three electrode types are as follows: unmodified: 124 mV, Smooth Au@Pt: 72 mV, and Hairy Au@Pt: 74 mV. These values are indicative of improved kinetics on the modified electrode surfaces. Based on these voltammograms, the Randles-Sevcik equation was employed to calculate the electrochemically active surface area (EASA), and the values for the unmodified, Smooth Au@Pt NRs, and Hairy Au@Pt NR-modified electrodes are the following, respectively: 4.59, 5.29, and 5.34 mm^2^.

Finally, Figure 4d displays the EIS Nyquist plots of all three electrode types measured against the Fe­(CN)_6_^3–^/^4–^ redox couple. The polarization resistance (Rp), representing the interfacial resistance between the electrodes and the electrolyte, was analyzed to investigate the behavior of each electrode. The plots demonstrated an excellent fit to a constant phase element (CPE) with a diffusion model circuit (Figure S3).

The CPE behavior is often associated with the irregular topography of solid electrodes, which causes variability in properties such as the solution resistance, interfacial capacitance, and localized current density. This effect arises from a complex balance between rapid diffusion processes and slower kinetic mechanisms that regulate the adsorption of ions or other species from the electrolyte onto the electrode surface. Studies have consistently shown that as the electrode surface becomes rougher, the CPE reflects a more uniform response. This observation implies that greater surface area reduces the relative influence of adsorbed ions or impurities, leading to a more homogeneous electrochemical interface.^25,36^

The unmodified electrode (cream curve) shows a semicircle with a diameter (Rp) of 825 Ω. Whereas for both modified electrodes, this value is almost identical averaging approximately at 73 Ω (blue and pink curves). This significant decrease in polarization resistance indicates that the much rougher surface of the modified electrode facilitates more favorable electron transfer from the electrolyte (electrochemical double-layer) through facilitating much higher surface areas.

#### Electrochemical Characterization of H_2_O_2_

H_2_O_2_ can oxidize via two mechanisms. The first involves an electron-loss pathway (eq 2), commonly reported in electrochemical oxidation processes.^37^ The second is a nonfaradaic oxidation process (eq 3), which is exothermic and typically observed on noble metal surfaces such as Pt, its alloys, or enzymatic systems.^38^ The dominant pathway is likely influenced by a combination of particle characteristics and environmental conditions, both of which impact analytical performance and sensitivity toward H_2_O_2_.^39^

2 $$H_{2}O_{2}\to O_{2}+2H^{+}+2e^{-}$$

3 $$2H_{2}O_{2}\to {2H}_{2}O+O_{2}$$

The obtained CV peaks only show the anodic peak (*E*_*Pa*_) on the modified electrode’s surface—appearing at approximately 560 mV vs Ag/AgCl on the Smooth Au@Pt modified electrode (Figure S4a) and shifting to around 515 mV on the Hairy Au@Pt modified electrode (Figure S4b). This shift suggests that the surface morphology of the NRs affects the oxidation kinetics with the Hairy Au@Pt configuration facilitating H_2_O_2_ oxidation more effectively (Figure 5a).

**Figure 5:**
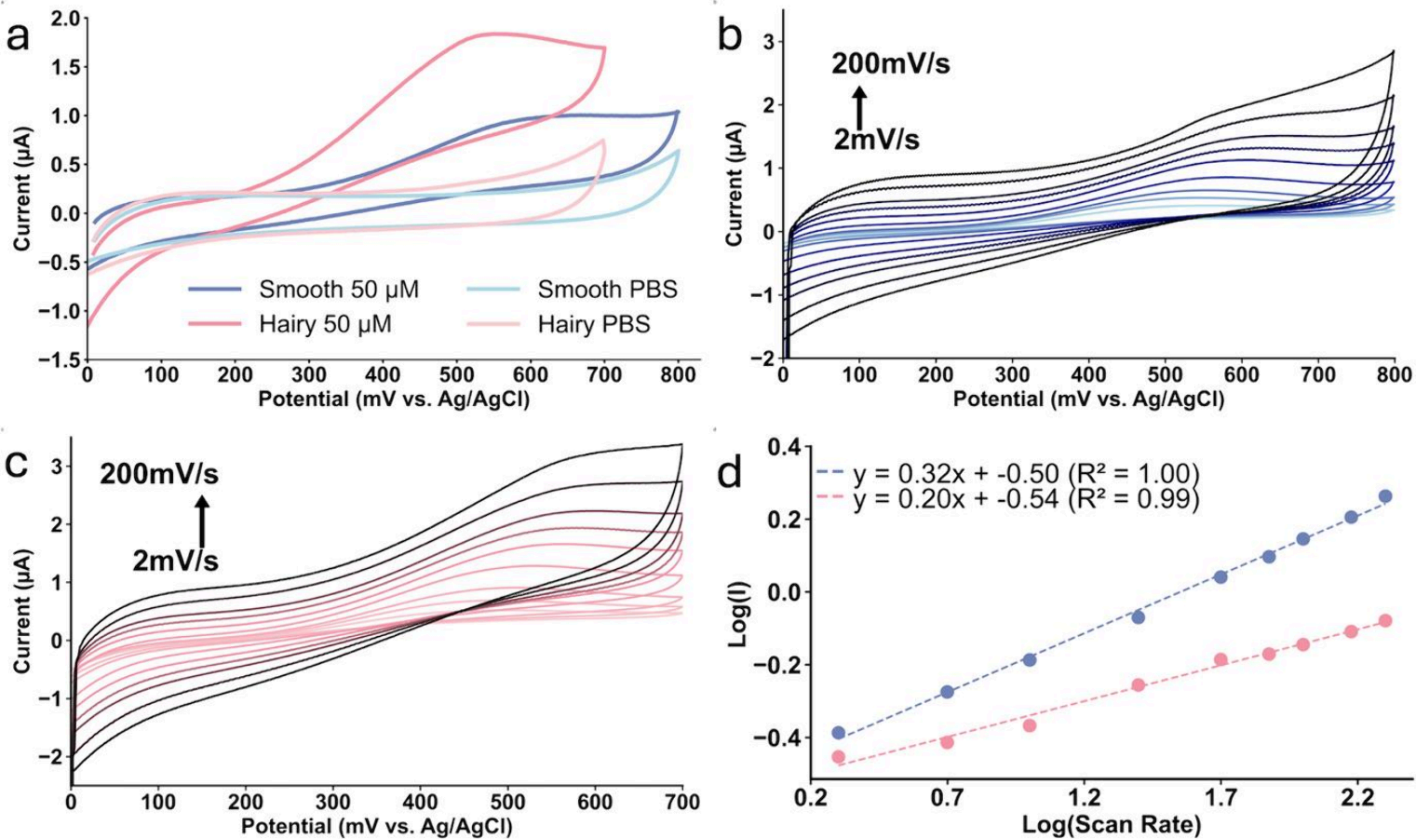
Cyclic voltammograms of (a) comparison peaks of the Smooth Au@Pt (light and dark blue) and Hairy Au@Pt (light and dark pink) electrode vs PBS and 50 μM of H_2_O_2_/PBS with a scan rate of 50 mV·s^–1^. Effect of scan rates between 2 to 200 mV·s^–1^ for (b) Smooth Au@Pt and (c) Hairy Au@Pt NR-modified electrode vs 50 μM of H_2_O_2_/PBS. (d) The logarithm of peak current is vs the logarithm of the scan rate for Smooth Au@Pt (blue) and Hairy Au@Pt NRs (pink) modified electrode.

While both mechanisms result in H_2_O_2_ oxidation, their relative presence and dominance appear to be determined by the structural and chemical properties of the catalytic interface. As the XPS analysis showed in the previous section, the Hairy Au@Pt electrode predominantly contains metallic Pt^0^, whereas the Smooth Au@Pt surface has a mixture of Pt^0^ and Pt^2+^. Although Pt^0^ is in a low formal oxidation state, it can function as a catalyst and still support H_2_O_2_ oxidation through mechanisms that may not require direct electron transfer from the metal.

It is likely that nonfaradaic oxidation is preferentially present on metallic surfaces that remain electronically stable and do not undergo redox transitions during the reaction. Specifically, Pt^0^ despite being in a reduced oxidation state, could facilitate this catalytic pathway by providing a high density of electronic states near the Fermi level indicative by the lower oxidation potential, which may allow it to stabilize reaction intermediates without itself being oxidized.^40,41^ It may be that the adsorbed H_2_O_2_ molecules cleave their O–O bond on Pt^0^ surfaces, forming intermediates such as Pt–OOH or Pt–OH, which are subsequently oxidized or decomposed under applied potential—without requiring oxidation of the Pt.^42–44^

Following this, the low abundance of Pt^2+^ on the Hairy Au@Pt surface may reduce the probability of catalyst deactivation through surface poisoning or intermediate trapping, which are commonly associated with oxidized Pt species like PtO or Pt­(OH)_2_.^45^ These species may stabilize less reactive forms, such as HOO^–^ or hinder the release of products, thereby limiting catalytic turnover. In contrast, a Pt^0^-rich surface is more likely to offer better accessibility to active sites, supporting more efficient catalysis.

The CVs were studied in biological concentrations ranging from 500 nm to 50 μM. Although the Smooth Au@Pt modified sensor could not measure less than 1 μM of H_2_O_2_ in comparison to Hairy Au@Pt detecting 500 nM. It is notable to mention when H_2_O_2_ concentration increases by 5 μM and then to 50 μM, there is noticeable positive shift in the peak potentials. This can be attributed to H_2_ production in the cell and the changing pH values. Additionally, it is important to highlight the lack of response and the near-complete insensitivity of the unmodified electrode surface toward H_2_O_2_, as demonstrated in Figure S5. Based on the presented data and the considerations regarding the oxidation pathway, we propose that the enhanced H_2_O_2_ oxidation observed at the Hairy Au@Pt electrode arises from a greater contribution of the nonfaradaic mechanism. Although Pt^0^ may not engage directly in electron transfer, it has been shown to have a strong catalytic role in stabilizing intermediates and enabling chemical oxidation under applied potential in various chemical and electrochemical reactions.^46,47^ This could explain the improved sensitivity compared to the Smooth Au@Pt electrode, where Pt^2+^ may facilitate faradaic oxidation but also lead to passivation and reduced catalytic performance.

To investigate the nature of H_2_O_2_ interactions occurring on the electrode surface, we studied the effect of varying the scan rate on the peak current values (Figure 5b and 5c). By increasing the scan rate, an increase in peak current on both Smooth and Hairy Au@Pt modified electrodes can be seen vs 50 μM H_2_O_2_/PBS. In Figure 5c, the logarithm of peak current is plotted against the logarithm of the scan rate, providing insights into the nature of the electrochemical interactions. A slope within the range of 0.2 to 0.6 indicates a diffusion-controlled process, while a slope between 0.75 and 1 suggests an adsorption-controlled process. Slopes between 0.6 and 0.75 imply a combination of both diffusion and adsorption control.^48^ Based on this analysis, we conclude that in the presence of H_2_O_2_ on both Smooth and Hairy Au@Pt modified electrodes (Slopes 0.32 and 0.20, respectively), the electrochemical mechanisms are primarily governed by diffusion-controlled processes.

#### Effect of O_2_

When H_2_O_2_ is saturated with O_2_, a significant change in the current behavior can be observed in both modified electrodes (Figure 6 with representative samples). This phenomenon is likely due to the catalytic activity of Pt^0^ in promoting the reduction of oxygen, which occurs within a similar potential range.^49^ With Pt facilitating oxygen reduction reactions, there is a chance of potential overlap with H_2_O_2_. Therefore, if this electrode should be employed in biological matrices, fluctuations in oxygen concentration are expected to influence the electrochemical response, which in turn requires further potential adjustment. Based on the current profile, the Hairy Au@Pt modified electrode exhibits greater stability when compared to the Smooth Au@Pt where the current shows no measurable peaks.

**Figure 6:**
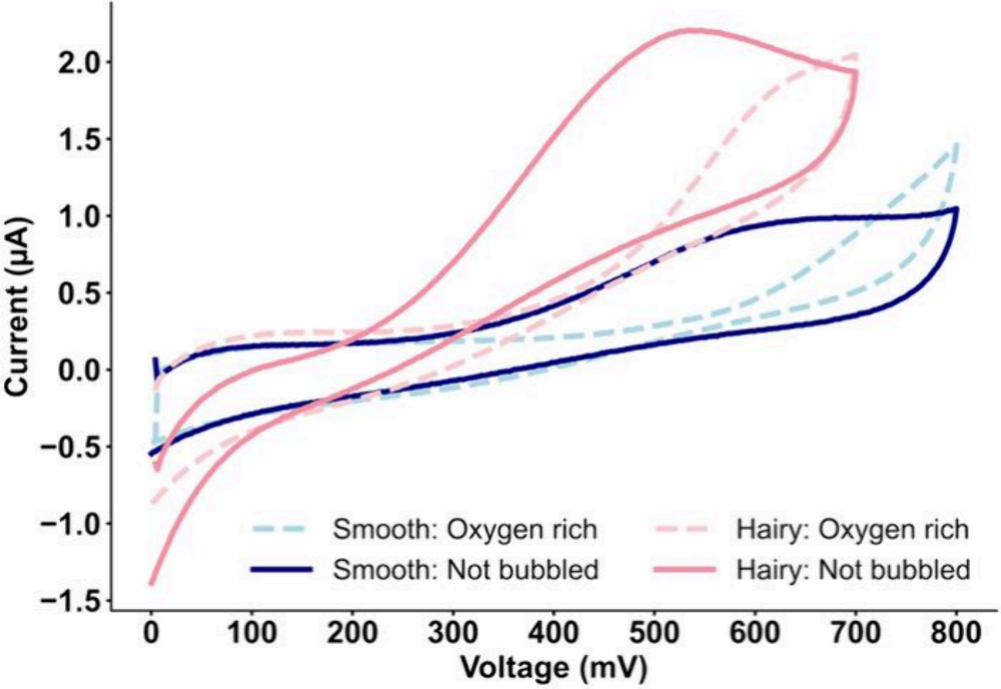
Cyclic voltammograms of 50 μM H_2_O_2_/ PBS on Smooth and Hairy Au@Pt NRs in the absence (solid lines) and presence (dashed lines) of O_2_ bubbling, scan rate = 50 mV·s^–1^.

#### CA Determination of H_2_O_2_

Figure S6a and S7a show the CA responses of Smooth and Hairy Au@Pt modified electrodes in the potential optimization process. An increase in the applied potential corresponded to an increase in current for both Smooth and Hairy Au@Pt electrodes. This trend remained consistent, except for the Hairy Au@Pt electrode at 500 mV, which is near the oxidation potential observed in CV peaks, indicating maximal molecular activity at this potential.

Additionally, after normalizing the response obtained in different potentials, S/N ratio was calculated (see Figure S6b and S7b). The results revealed that the Hairy Au@Pt electrode achieved the highest S/N ratio at 500 mV. However, the Smooth Au@Pt electrode showed negligible differences in S/N ratio between 500 and 600 mV. Consequently, for both electrodes, 500 mV was selected as the optimal potential to ensure reliable and comparable results.

The CA responses of the Smooth and Hairy Au@Pt modified electrodes (Figure 7a and 7b) to different concentrations of H_2_O_2_, spanning from 500 nM to 50 μM, are presented below. The Smooth Au@Pt electrode exhibits a measurable current response starting at 1 μM. In contrast, the Hairy Au@Pt electrode exhibits a lower limit of quantification by detecting concentrations down to 500 nM, half the minimum detectable concentration of the Smooth Au@Pt electrode. Furthermore, at a concentration of 1 μM, the current intensity measured with the Smooth Au@Pt electrode is 78% of the current observed with the Hairy Au@Pt electrode at half of the concentration (500 nM), underscoring the significantly enhanced sensitivity of the Hairy Au@Pt electrode. The calibration curves, shown in Figure 7c, are described by the equations I (μA) = 0.012­[H_2_O_2_] (μM) + 0.092 (r = 0.9975, n = 5) for the Smooth Au@Pt electrode, and I (μA) = 0.026­[H_2_O_2_] (μM) + 0.087 (r = 0.996, n = 4) for the Hairy Au@Pt electrode.

**Figure 7:**
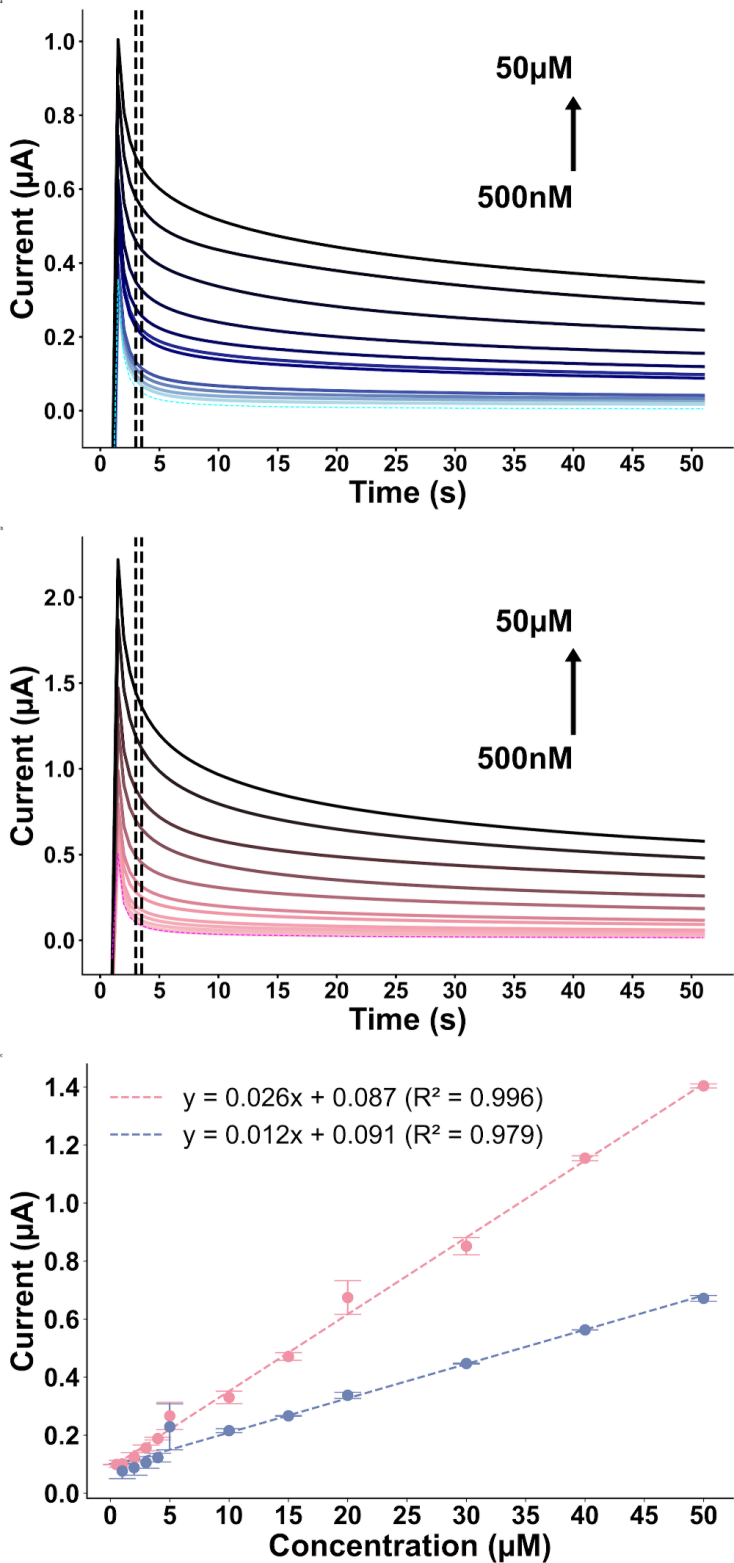
Amperometric response of (a) Smooth Au@pt NRs and (b) Hairy Au@Pt NRs in PBS (dashed lines), in concentrations between 1–50 μM and 500 nm–50 μM H_2_O_2_/PBS, respectively, potential step = 200 to 500 mV. Calibration curves of (c) Smooth (Blue) and Hairy (Pink) Au@Pt NRs according to the amperometric data.

Analysis of the averaged data points between 3 and 3.5 s reveals that the Smooth Au@Pt electrode operates within a narrower linear range of 1 μM to 50 μM and exhibits a lower sensitivity of 0.170 μA μM^–1^ cm^–2^. In comparison, the Hairy Au@Pt electrode maintains a broader linear response from 500 nM to 50 μM with a sensitivity of 0.368 μA μM^–1^ cm^–2^. Additionally, based on signal-to-noise ratios, the LODs are calculated to be 370 nM for the Smooth Au@Pt electrode and 189 nM for the Hairy Au@Pt electrode, thereby clearly lower for the Hairy Au@Pt electrode and further underscoring its enhanced analytical capabilities. The reproducibility of Hairy and Smooth Au@Pt NR-modified electrodes was assessed by CA using three independently prepared electrodes. Under identical conditions (50 μM H_2_O_2_/ PBS; averaged data points between 3 and 3.5s acquisition window), the relative standard deviation (RSD%) of the current response was 2.32% for the Smooth and 5.78% for the Hairy Au@Pt configuration.

The enhanced performance of Hairy Au@Pt NRs in detecting H_2_O_2_ can be traced to a combination of their tailored surface structure, stable electronic properties, and the dynamic behavior of their most reactive features—particularly the edges. While surface area and roughness are important, it is the quality of the exposed sites, not just their quantity, that drives the significant increase in catalytic activity. And at the heart of that lies the critical role of edge structures.

Smooth Au@Pt NRs are dominated by flat, low-index crystal surfaces that are more stable but less chemically active. These broad, ordered planes contain atoms that are tightly packed and energetically satisfied, making them less likely to interact strongly with H_2_O_2_ molecules or assist in breaking their bonds. Only a small fraction of the surface—primarily at the corners and edges—offers more reactive sites, but in these smoother structures such features are limited and contribute minimally to overall catalytic behavior.

Hairy Au@Pt NRs, in contrast, show a noticeable shift in the surface architecture. Their small (2–4 nm) bumps provide a highly textured surface with significant curvature and topological complexity. This morphology significantly increases the presence of edge-like environments and high-index facets.^50^ These high-index surfaces are composed of atoms in more strained, undercoordinated positions, which are far more chemically active.

However, it is the edges themselves—sharp steps, corners, and atomic kinks—that play a uniquely powerful role in catalysis. These sites are not simply more active; they are fundamentally different in how they interact with the reactant molecules. Edge atoms have a distinct electronic structure compared to flat surfaces, often exhibiting a higher local charge density and orbital flexibility. This makes them ideal for attracting and activating molecules like H_2_O_2_. Additionally, edges promote stronger binding of key intermediates during oxidation and facilitate the transfer of electrons from the surface to the electrode. They act as catalytic hotspots where the reaction is not only more likely to occur, but also proceeds more efficiently.^23,51^

Additionally, these edge structures are not static. Under reaction conditions, they can evolve—reshaping or reorganizing to expose even more reactive configurations.^52–54^ This adaptability means the surface of Hairy Au@Pt NRs does not just provide one form of active sites; it actively responds to the reaction environment, maintaining or even enhancing its reactivity over time. The ability to direct the formation of such edge-rich features through synthesis, as shown in this system, is a powerful strategy in catalyst design.

Supporting this, XPS results show that Pt in Hairy Au@Pt NRs remains in the metallic Pt^0^ state, which is important for maintaining high conductivity and efficient charge transfer. This stable electronic environment works hand-in-hand with the edge structures, ensuring that the flow of electrons during H_2_O_2_ oxidation is not bottlenecked by surface degradation or electronic traps.

Although these nanorods produce a slightly higher background current, likely a result of their greater surface area and capacitive behavior, the faradaic response to H_2_O_2_ is dramatically stronger. This is especially beneficial in biological and low-concentration settings, where high sensitivity and a fast response are essential.

### Cell Viability Test

We investigated the effect of different types of nanoparticles on the viability of neuroblastic cells. For these nanoparticles to be suitable as sensor modifiers in a hypothetical neural implant, they must not significantly compromise the survival of the neural cells. Several rounds of testing for the sensor’s stability in protein rich biological fluids that typically surrounds cells were carried out; however, the NRs do not demonstrate adequate adhesivity to the GCE surface in the mentioned situation. That is why the cytotoxicity was carried out by placing the particles directly on the cells.

The data demonstrates that increasing concentrations of NR solutions result in a noticeable reduction in cell viability which is in line with previous studies on the subject^55,56^ (see Figure 8). As the concentration of NRs increases, the percentage of viable cells decreases, although this effect is not uniform across the two types of NRs tested. For Smooth Au@Pt NRs, cell viability at a concentration of 2.5 μL is approximately 89.1%, decreasing to 86.4% at 7.5 μL. In contrast, Hairy Au@Pt NRs reduce viability from 76.3% at 2.5 μL to 68.9% at 7.5 μL. μn average, cells exposed to Smooth Au@Pt NRs exhibit 18% higher viability compared to those exposed to Hairy Au@Pt NRs. The surfactant, CTAB, is toxic to cells, shown with the spiked surfactant solutions reducing cell viability drastically to approximately 10%. We attempted to remove the remaining CTAB via centrifugation. However, trace amounts of this material likely remain. The biohazardous nature of CTAB, combined with the complex structure of Hairy Au@Pt NRs, suggests that these particles may trap residual surfactants and other chemicals more effectively than Smooth Au@Pt NRs. This matter was also seen in the HR-TEM imaging results.

**Figure 8:**
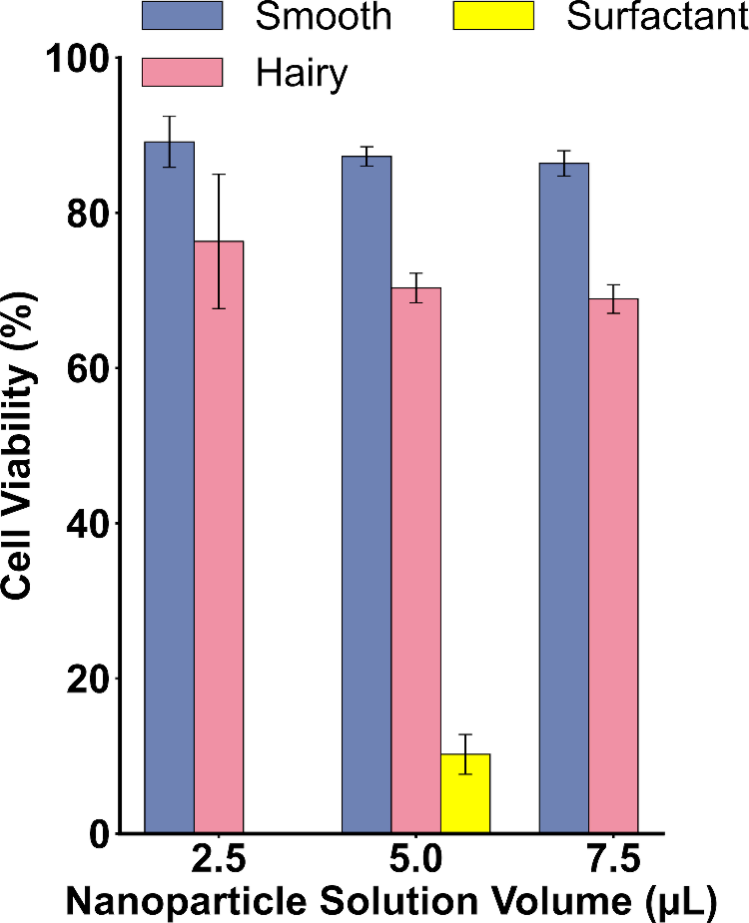
Viability of cells in the presence of Smooth and Hairy Au@Pt NRs (blue and pink) and surfactant solution (yellow).

Although the cell-viability data and intrinsic stability of the particles are acceptable, the stability and composition of the electrode still need to be optimized for direct electrochemical measurements in cellular environments. A key improvement will be to introduce surfactant-removal procedures that minimize residual CTAB, thereby eliminating its adverse electrochemical effects, and to stabilize the nanorods within more favorable carbon materials.

### Comparison to Other Studies

A comparative evaluation is conducted between Smooth and Hairy Au@Pt NRs-modified electrodes and various other sensors modified with both Au and Pt in different configurations, all designed for H_2_O_2_ detection. The performance of these electrodes is detailed in Table 1, which highlights key parameters, such as linear range, detection limit, and sensitivity. The table is purposefully designed to include only Pt- and Au-based sensors. It is noteworthy that some of the reported linear ranges do not correspond to typical biological concentration limits, as several studies have been detecting H_2_O_2_ in nonbiological compounds. Also, most of the sensitivity values in the list are based on the theoretical surface areas. Due to the different methods of electrode modifications, making a fair comparison between the present particles and their quantities might be challenging. While the charge transfer resistance and the EASA are generally inversely related, where a larger EASA typically enhances charge transfer by providing more space for electrochemical reactions, this relationship is not always straightforward. Surface roughness can complicate this dynamic and the real surface area calculation. If the surface features are smaller than the diffusion layer thickness, then the EASA may increase without a corresponding improvement in reaction rates. This makes the relationship between electron transfer and EASA less predictable, emphasizing the need to account for surface roughness in evaluating electrochemical performance.

**Table 1:** Comparison of the Electrochemical Significant Values for the Smooth and Hairy Au@Pt NRs Modified to Other Studies Incorporating Gold and Platinum Nanoparticles as Modifiers

Electrode materials	Linear range (μM)	LOD (μM)	Sensitivity (μA μM^–1^ cm^–2^)	*E*_Ag/AgCl_ (mV)	Response time (s)	Reference
AuPt/ZIF-8-rGO	0.1–18000	0.019	-	–100	-	^57^
AuPt/Ce-MOF/CFC	0.15–214.95	0.08	-	500	<8	^58^
Au@Pt NPs/ITO	0.5–1000	0.11	-	–200	<60	^59^
PtAu/G-CNTs	2–8561	0.6	0.313	–470	<4	^60^
Au_1_Pt_2_/SPCE	2.5–5000	4.8	0.155	–320	<120	^61^
SiO_2_/APTMS/AuPt	5–72000	2.6	0.047	–200	5	^62^
RGO/nAuPtAMSs	5–4000	0.008	1.117	–500	<5	^63^
Pt_0.5_Au_0.5_@C	7–6500	2.4	0.210	300	-	^64^
**Smooth Au@Pt NRs**	**1–50**	**0.370**	**0.170**	**560**	**<5**	**This work**
**Hairy Au@Pt NRs**	**0.5–50**	**0.189**	**0.368**	**515**	**<5**	

The detection limits observed for both the Smooth and Hairy Au@Pt NRs-modified electrodes rank among the lower values in Table 1. Additionally, the sensitivity of the electrodes fabricated in this study surpasses those of most other examples. Given its favorable analytical performance, the Hairy Au@Pt NRs-modified electrode shows strong potential for the development of highly effective H_2_O_2_ sensors.

## Conclusion

In this work, we investigated how the shape of the Au@Pt nanoparticles influences the electrochemical performance of a H_2_O_2_ sensor. We demonstrated that structural modifications, particularly increased surface area, edge site exposure, and facet orientation, lead to significant improvements in sensitivity and selectivity.

Crucially, our study also highlighted the roles of the oxidation state and the effective mass of active material in contact with the analyte. While many existing approaches emphasize compositional tuning, they often overlook the interplay between structural heterogeneity, redox behavior, and material loading. We showed that even subtle variations in these parameters can markedly affect electrochemical responses, including the reaction kinetics and current density.

By systematically isolating these effects, we underscored the need to move beyond the assumptions of nanoparticle uniformity. Our findings reveal that both morphology and physicochemical state play critical roles in sensor efficiency, with direct implications for performance in biological environments, where factors such as cytotoxicity and cellular interactions are sensitive to nanoparticle structure and oxidation chemistry. This connection was confirmed by detailed morphological analyses using (HR-)­TEM, UV–vis spectroscopy, and XPS.

In the process of fabricating these ∼40 nm in length NRs, by using a gold core with a thin platinum shell, we not only reduced the overall use of platinum, a critical raw material, but also enhanced catalytic performance. This design is especially advantageous given the scarcity and high cost of platinum.

H_2_O_2_ detection was performed at concentrations typical of biological systems (1–50 μM), highlighting the sensor’s potential for future in vitro or in vivo implant applications. Various electrochemical characterizations (such as EIS and ISR and OSR probe studies) further confirmed the impact of nanoparticle shape on sensor performance. The structure that possessed a higher surface area and a lower oxidation state (Hairy Au@Pt NR-modified electrode) demonstrated a more favorable kinetical behavior, that can be seen in the *E*_*Pa*_ values when compared to that of the Smooth Au@Pt NR-modified electrode (H_2_O_2_ oxidation potential 515 vs 560 mV).

This study was conducted with future applications of sensors for neurotransmitter detection in mind. While CV revealed differences among the modified electrodes, CA was selected as the primary detection method. The neural H_2_O_2_ is a byproduct of the l-glutamate oxidase reaction in the body, and the enzymatic reaction is highly selective. CA is particularly suited for detecting electroinactive species, such as glucose and glutamate, which can be measured indirectly via oxidase enzymes that generate H_2_O_2_.

The CA results demonstrated sensitive responses in under five s following potential optimizations. In the final outcome, we observed that Hairy Au@Pt NR-modified electrode limit of detection is nearly the half (0.189 vs 0.370 μA) and its sensitivity is almost double (0.368 vs 0.170 μA μM^–1^ cm^–2^) the numbers of Smooth Au@Pt NR-modified electrode versus H_2_O_2_.

Preliminary cell toxicity and viability tests showed that the nanoparticles are biocompatible, with over 70% of cells surviving on average. This encouraging result paves the way for further cell-based studies and biomedical applications.

Overall, our work demonstrates that careful control over nanoparticle shape not only improves sensor performance but also supports a sustainable and biocompatible approach for developing biosensors and implantable devices.

As the next step, while we have performed H_2_O_2_ detection as a model reaction to evaluate electrochemical responsiveness, the ultimate goal of this sensor platform is to integrate glutamate oxidase onto a carbon-based material in future work. The strategy is to detect glutamate indirectly through enzymatically generated H_2_O_2_, which results from the oxidation of glutamate catalyzed by glutamate oxidase. This approach relies on a well-established enzymatic mechanism in which the enzyme serves as a highly specific biorecognition element for glutamate.

## Supplementary Material



## Data Availability

Data for this article, including raw data, is available on Zenodo (https://zenodo.org/records/16090859).

## Notes

Fabrication method for Au@Pt NRs, TEM images of Au NRs, Smooth and Hairy Au@Pt NRs in several magnifications, XPS survey spectra of Au@Pt NRs, Equivalent circuit of constant phase element with diffusion resistance, cyclic voltammogram of Smooth and Hairy Au@pt NRs vs H_2_O_2_/PBS in their respective linear ranges, cyclic voltammogram of the unmodified electrode vs PBS and 50 μM H_2_O_2_/PBS, amperometric response and its S/N ratio for shifting oxidation potentials for the Smooth and Hairy Au@Pt NR-modified electrode vs H_2_O_2_ 50 μM/PBS (PDF)

## Abbreviations

CA: Chronoamperometry
CPE: Constant phase element
CV: Cyclic voltammetry
EASA: Electrochemically active surface area
EIS: Electrochemical impedance spectroscopy
HR-TEM: High resolution transmission electron microscopy
ISR: Inner-sphere redox
LOD: Detection limit
NP: Nanoparticle
OSR: Outer-sphere redox
PBS: Phosphate buffer saline
TEM: Transmission electron microscopy
XPS: X-ray photoelectron spectra
