# Supplementary material for: Various Configurations of Au@Pt Nanostructures on Modified Electrochemical Sensors for H2O2 Detection
Source: ACS Appl Nano Mater. 2025 Jul 22;8(30):15382–94. doi: 10.1021/acsanm.5c03116 (PMC12322878; doi:10.1021/acsanm.5c03116)
Supplement: Supplementary file 1 [file an5c03116_si_001.pdf]

Supporting Information

Various Configurations of Au@Pt Nanostructures on Modified Electrochemical Sensors for H_2O_2 Detection

Bahar Mostafiz [a], Johanna Suni [a], Edna De Jesus Cabrera [a], Nidhin George Mathews [b], Rituporn Gogoi [a], Gaurav Mohanty [b], Vipul Sharma [a], Emilia Peltola [a*]

a. Department of Mechanical and Materials Engineering, University of Turku, Turku, FI-20014, Finland
b. Materials Science and Environmental Engineering, Faculty of Engineering and Natural Sciences, Tampere University, Tampere, FI- 33014, Finland

* Corresponding author, Email: Emilia.peltola@utu.fi

Fabrication method for Au@Pt NRs

Au NRs were synthesised in a non-seeding approach. An aqueous CTAB solution (8.33 mL, 180 mM) was combined with NaCl (225 μL, 0.1 M), $HAuCl_4.3H_2O$ (180 μL, 0.05 M), and $AgNO_3$ (180 μL, 0.01 M) solutions. This mixture turns to a yellowish-brown hue. Upon gentle shaking, 180 μL AA 0.1M was introduced and inverted for 30 seconds to mix, leading to a color shift from yellowish-brown to transparent. Subsequently, ice-cold $NaBH_4$ (10 μL, 3.14 mM) was added, followed by another 30-second inversion, which resulted in a purple solution. The solution was then left undisturbed at ambient temperature for at least 12 hours. Post-synthesis, the samples were transported to a 15 mL falcon tube, placed in 30 degrees Celsius water for the sedimented surfactant to change from crystalline structure to dissolved, and then underwent thrice centrifugation (11000 rpm for 10 minutes each time) and washing with MiliQ water to eliminate surplus reactants and were concentrated by to a final volume of 7 ml.

The Au@Ag NRs were then produced using a PVP-based method to ensure colloid stability. A mixture of the Au NR solution (0.8 mL) with a PVP solution (4 mL, 1% w/w) was made, followed by the addition of $AgNO_3$ (180 μL, 0.001 M) and AA (100 μL, 0.1 M), with careful stirring after each addition. Then by introducing NaOH (200 μL, 0.1 M), a bluish-green color appeared, indicative of Au@Ag NRs formation.

For the Smooth Au@Pt NRs fabrication, galvanic replacement was employed using H_2SO_4. To the Au@Ag NR solution (5 mL), K_2PtCl_4 (250 μL, 0.001 M) and H_2SO_4 (50 μL, 0.02 M) were incorporated. The reaction mixture was magnetically stirred at 300 rpm and maintained at 40°C for 5 hours. In the case of Hairy Au@Pt NRs synthesis, K_2PtCl_4 (250 μL, 0.001 M) was added to the Au@Ag NR solution (5 mL). This solution was agitated magnetically at a faster pace of 500 rpm and a higher temperature of 60°C for 5 hours.

The nanorods were separated by centrifugation at 7000 rpm for 20 minutes and washed multiple times with MiliQ water to purify.

The purpose of drop-casting equal volumes of both solutions onto the same glassy carbon electrode is to maintain a consistent nanoparticle count within the defined area. Since the stoichiometric values are identical, the primary difference lies in the pH level at which galvanic replacement occurs, which promotes the growth of the Hairy structures. This method ensures an equal concentration of nanoparticles from each solution, enabling a direct comparison of active material content. Consequently, any observed differences in electrochemical behaviour can be attributed to variations in nanoparticle composition rather than differences in deposition volume.

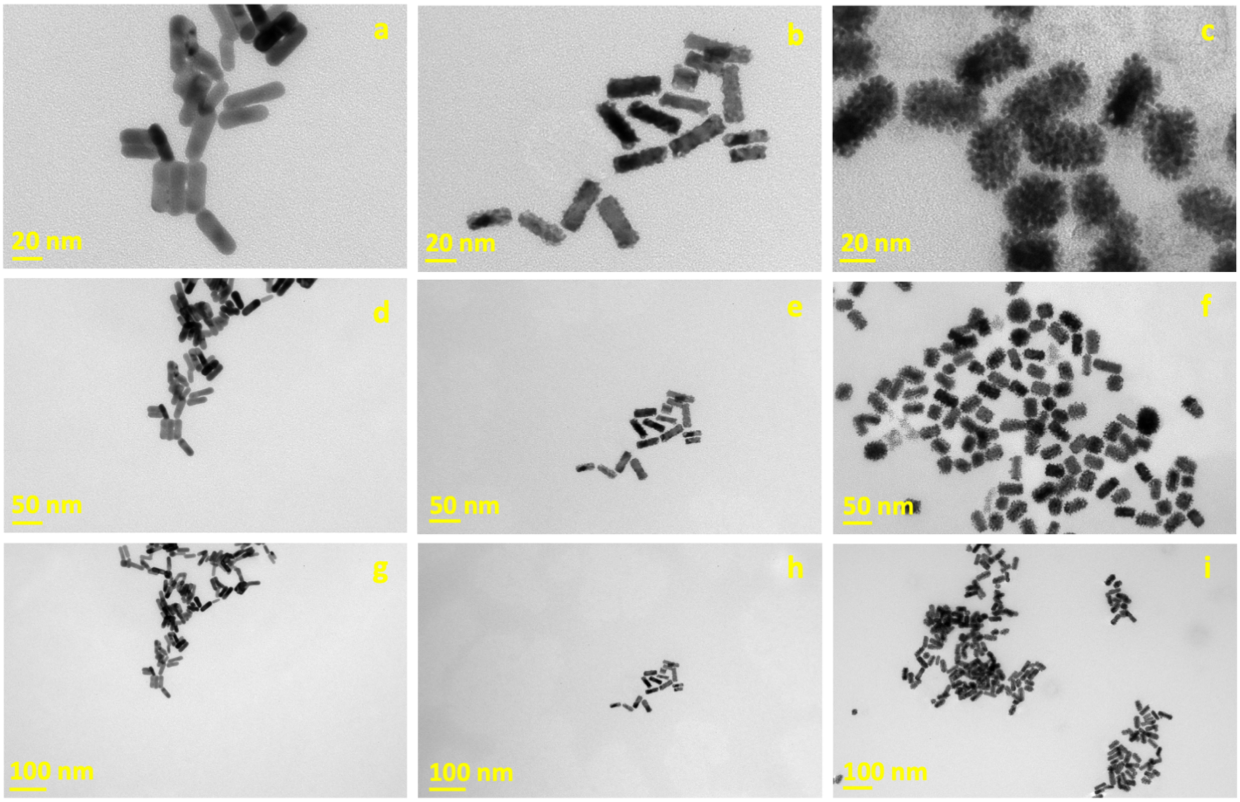

Figure S1. TEM images of Au NRs (a,d,g), Smooth Au@Pt NRs (b,e,h), and Hairy Au@Pt NRs (c,f,i).

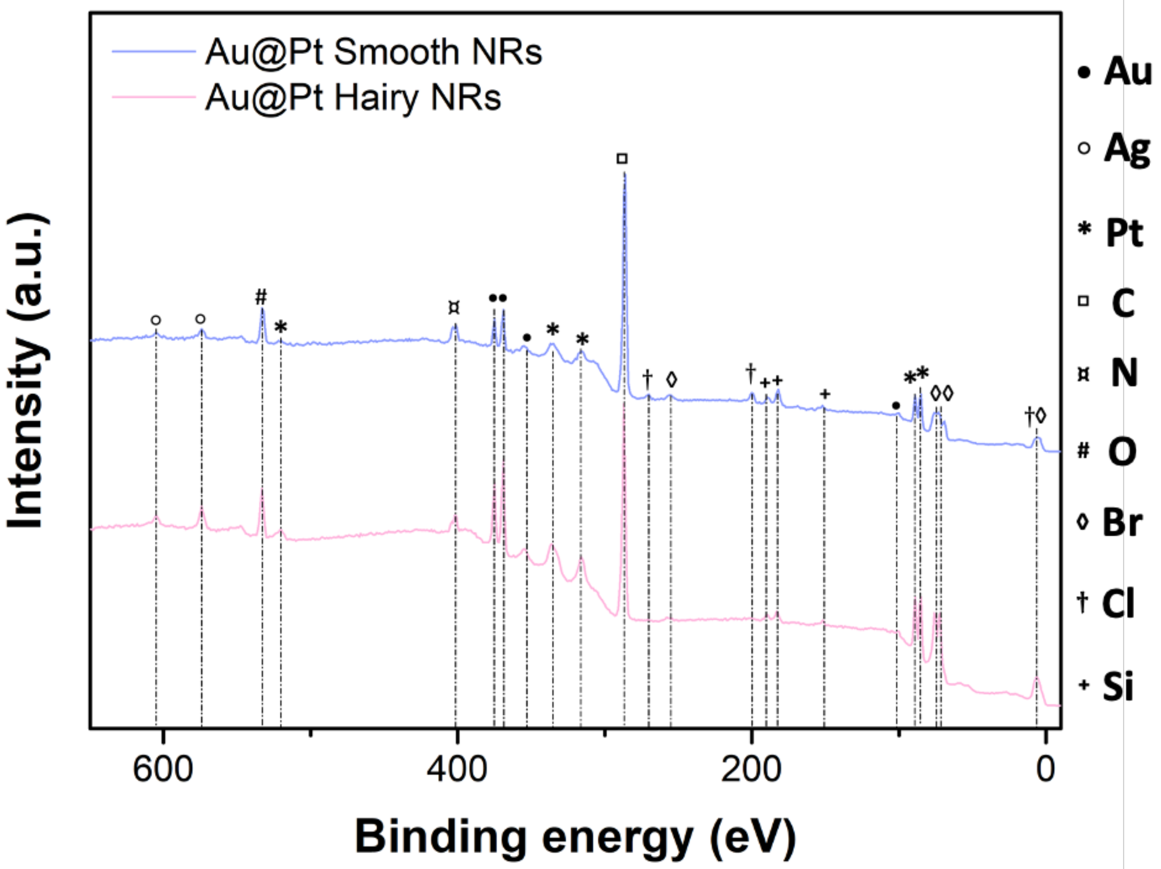

Figure S2. XPS survey spectra of Au@Pt NRs.

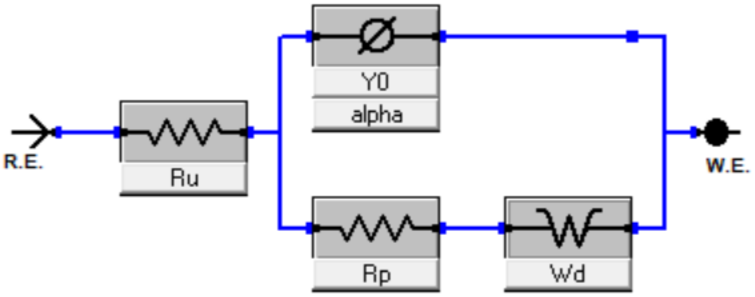

Figure S3. Equivalent circuit of constant phase element (CPE) with diffusion resistance.

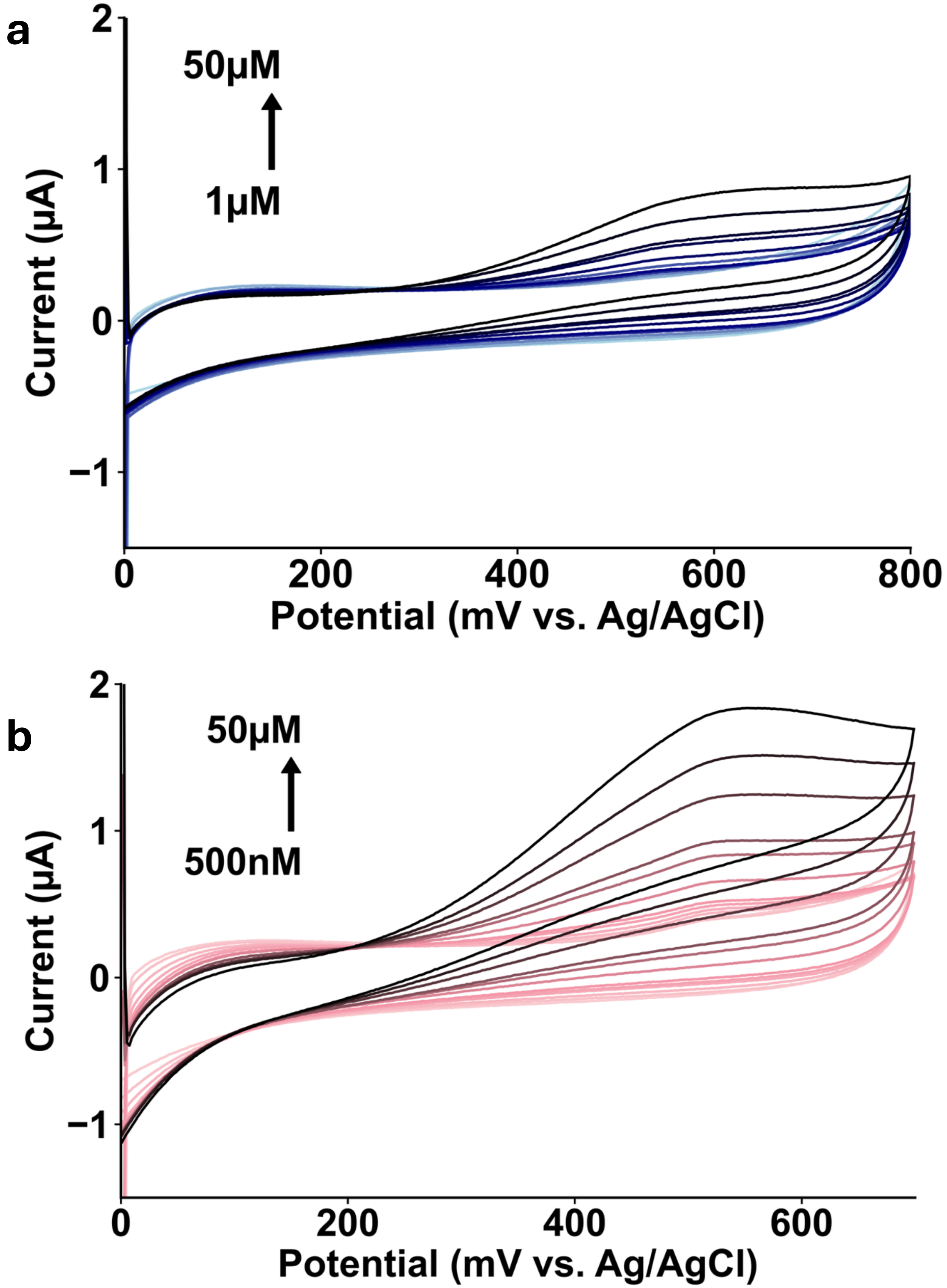

Figure S4. Cyclic voltammogram of (a) Smooth Au@pt NRs, (b) Hairy Au@Pt NRs in concentrations between 1µM-50µM and 500nm-50µM H_2O_2/PBS respectively in a scan rate of 50mV.s^{-1}.

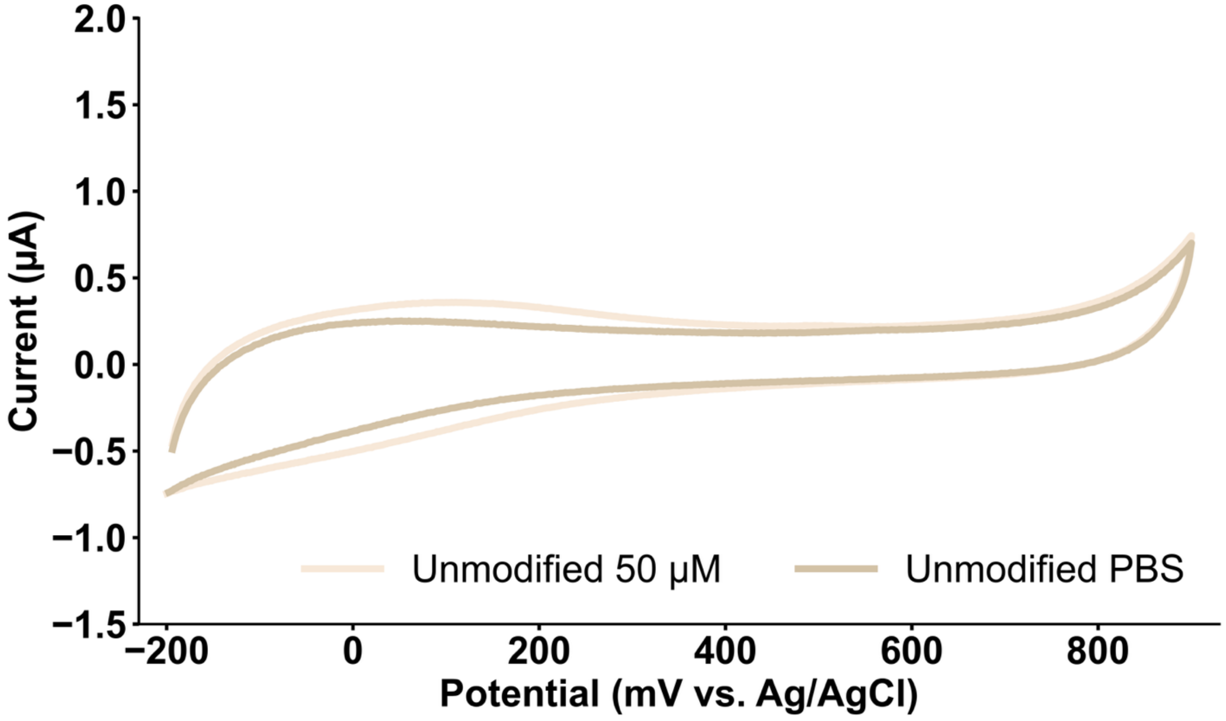

Figure S5. Cyclic voltammogram of the unmodified electrode in PBS and versus 50µM H_2O_2/PBS with a scan rate of $50mV.s^{-1}$.

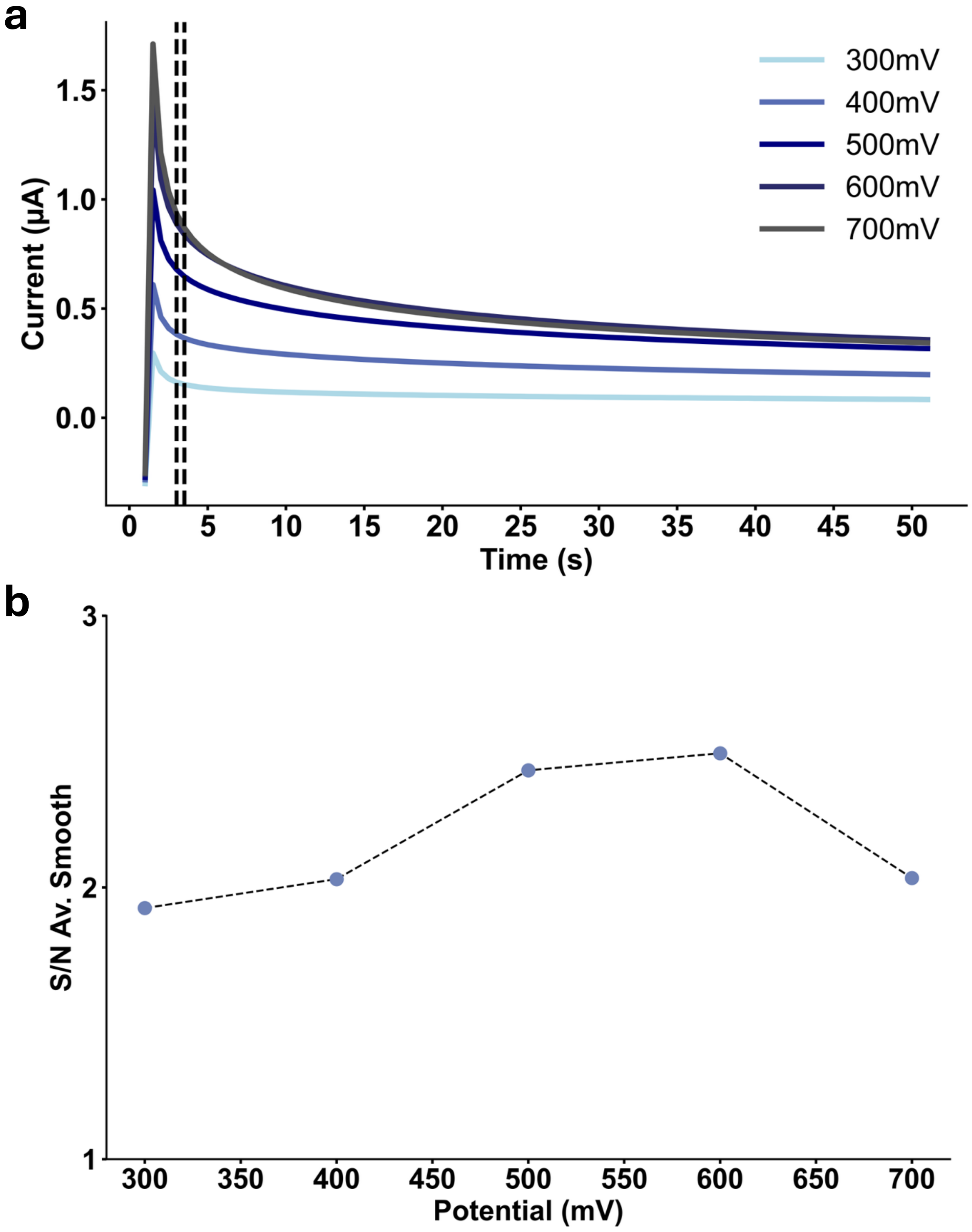

Figure S6. Amperometric response of the (a) Smooth Au@Pt NRs modified electrode vs. H_2O_2 50μM in PBS at pH 7.4 when the potential is shifting from 300 to 700mV. Effect of the ratio of signal to noise on the applied potential in (b) Smooth Au@Pt NRs modified electrode.

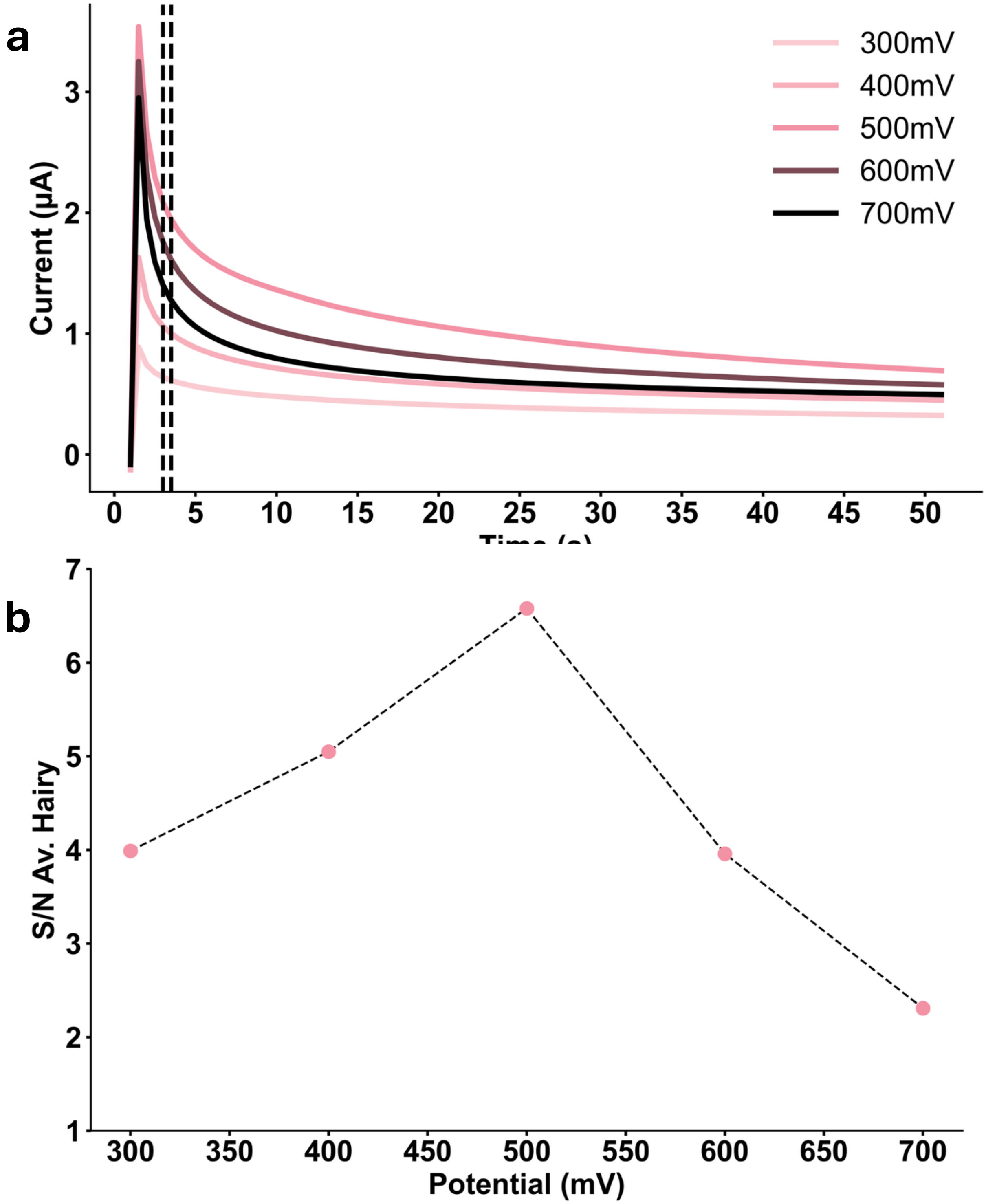

Figure S7. Amperometric response of the response of the (a) Hairy Au@Pt NRs modified electrode vs. H_2O_2 50μM in PBS at pH 7.4 when the potential is shifting from 300 to 700mV. Effect of the ratio of signal to noise on the applied potential in (b) Hairy Au@Pt NRs modified electrode.
